# Supplementary material for: Genome-Wide DNA Methylation and Gene Expression in Patients with Indolent Systemic Mastocytosis
Source: Int J Mol Sci. 2023 Sep 10;24(18):13910. doi: 10.3390/ijms241813910 (PMC10530743; doi:10.3390/ijms241813910)
Supplement: Supplementary file 1 [file ijms-24-13910-s001.zip › ijms-2575947-supplementary.pdf]

Supplementary materials

Table S1. Summary table of 85 CMRs.

deltaBeta	P.Value	CMR	gene	feature	cgi	chr	start	end	no.cpgs
0.3328	7.8249E-08	CMR1	HOOK2	Body	shore	chr19	12876846	12877188	4
0.2376	2.2321E-17	CMR2		IGR	opensea	chr1	225924665	225924683	2
0.1515	5.5525E-12	CMR3		IGR	shore	chr3	16924563	16924709	2
0.1456	1.4603E-09	CMR4		IGR	opensea	chr2	29179066	29179435	2
-0.1378	6.1083E-08	CMR5		IGR	island	chr22	49447845	49448320	3
0.1222	1.2099E-17	CMR6	INTS7	Body	opensea	chr1	212148758	212149423	2
0.1217	2.5599E-10	CMR7	SH3PXD2A	TSS1500	shore	chr10	105616094	105616523	2
0.1087	2.5023E-12	CMR8	TMPRSS6	Body	island	chr22	37493737	37494173	2
0.1047	1.4676E-11	CMR9	SGMS1	5'UTR	opensea	chr10	52134715	52135449	2
-0.1008	1.4746E-10	CMR10	APOB	TSS1500	shore	chr2	21267858	21268152	2
-0.0997	2.3214E-08	CMR11	RASGEF1B	5'UTR	shore	chr4	82392459	82392533	2
0.0986	4.7402E-11	CMR12		IGR	island	chr3	155027702	155027875	2
0.0957	3.0711E-09	CMR13	CDH7	TSS1500	shore	chr18	63416728	63417139	3
0.0938	4.1209E-08	CMR14		IGR	opensea	chr16	85455848	85456440	3
0.0895	1.9509E-14	CMR15		IGR	opensea	chr7	54794719	54794760	2
0.0880	3.5338E-11	CMR16	ADGRG6	TSS1500	shore	chr6	142621875	142622515	4
-0.0863	3.3584E-08	CMR17	LOC84931	Body	opensea	chr2	121223534	121223847	3
-0.0858	1.5291E-08	CMR18	KRTCAP3	TSS1500	shore	chr2	27664918	27666036	11
0.0798	8.5844E-09	CMR19	PAQR7	TSS1500	shelf	chr1	26198721	26199190	2
0.0791	2.9502E-08	CMR20	CCDC42	Body	opensea	chr17	8644134	8645026	2
0.0777	2.5646E-09	CMR21	FAM189A1	Body	opensea	chr15	29442040	29442169	2
0.0775	2.0624E-08	CMR22	SLC8A1	Body	opensea	chr2	40599929	40599951	2

-0.0769	8.0441E-12	CMR23	FUBP1	1stExon	island	chr1	78444801	78444909	4
0.0766	6.4584E-08	CMR24		IGR	shore	chr4	56023423	56024434	5
-0.0752	1.1735E-09	CMR25	C21orf91-OT1	Body	opensea	chr21	19157142	19157630	2
-0.0737	5.2954E-10	CMR26	FAM123C	TSS1500	shore	chr2	131512651	131512663	2
0.0729	1.3801E-08	CMR27	LOC100507195	TSS1500	opensea	chr12	68845785	68845935	2
0.0724	3.9684E-14	CMR28	LOC102724312	Body	shelf	chr1	1368387	1368846	3
0.0722	2.9806E-12	CMR29		IGR	shore	chr5	54515036	54515053	2
0.0719	2.9210E-09	CMR30	TCTEX1D1	Body	opensea	chr1	67241623	67241759	2
0.0713	4.7500E-09	CMR31	TMEM220	TSS1500	shore	chr17	10634349	10634532	3
-0.0690	5.3363E-08	CMR32	ANKMY1	5'UTR	island	chr2	241496830	241497599	8
-0.0685	4.5255E-09	CMR33	C9orf171	Body	island	chr9	135361993	135362480	3
-0.0680	1.8571E-09	CMR34	AFAP1	Body	island	chr4	7774727	7774768	2
0.0678	1.6169E-09	CMR35	COX19	Body	shelf	chr7	1012599	1012935	2
0.0677	6.9650E-09	CMR36	TMEM246-AS1	TSS1500	opensea	chr9	104230769	104231650	3
-0.0676	1.7234E-08	CMR37	GRM2	TSS1500	shore	chr3	51740622	51741473	9
0.0662	1.4129E-08	CMR38	RUNX3	Body	opensea	chr1	25240865	25240938	3
-0.0661	2.8580E-08	CMR39		IGR	island	chr19	384251	384408	3
0.0649	4.6826E-08	CMR40	TG	Body	opensea	chr8	134104425	134104479	2
-0.0644	1.7753E-10	CMR41	MFSD11	5'UTR	island	chr17	74733680	74733682	2
-0.0641	3.4287E-09	CMR42	SLC2A14	5'UTR	island	chr12	8025394	8025593	3
0.0638	4.3861E-09	CMR43	CDH4	Body	opensea	chr20	59878174	59878179	2
0.0637	4.0188E-16	CMR44	NEK6	3'UTR	opensea	chr9	127114252	127114588	2
-0.0637	8.5138E-09	CMR45	PLSCR2	TSS200	island	chr3	146187109	146187740	6
-0.0633	1.5764E-08	CMR46	PLEC1	Body	island	chr8	145003653	145005311	6
-0.0627	8.0848E-08	CMR47	PCDHGA4	Body	island	chr5	140821425	140821687	2
-0.0621	1.4659E-09	CMR48	CCER2	TSS200	opensea	chr19	39402823	39403047	6

0.0615	7.1388E-09	CMR49		IGR	shore	chr10	101282572	101283847	8
-0.0615	7.3134E-08	CMR50	GPR176	Body	shore	chr15	40211258	40211272	2
-0.0612	1.1385E-08	CMR51		IGR	opensea	chr14	61654132	61655862	4
0.0610	1.0215E-10	CMR52	PHKB	Body	opensea	chr16	47677874	47678468	2
-0.0603	2.5866E-09	CMR53	RAB22A	TSS1500	shore	chr20	56883532	56884425	4
0.0590	1.2735E-08	CMR54	SCG2	TSS200	opensea	chr2	224467340	224467359	2
-0.0584	7.9888E-08	CMR55		IGR	shelf	chr4	41872254	41872262	2
0.0584	1.7914E-09	CMR56	SH3GL2	Body	opensea	chr9	17601130	17601150	2
-0.0581	6.8818E-08	CMR57	RPS6KA2	Body	opensea	chr6	167004045	167004197	2
-0.0575	3.8766E-08	CMR58		IGR	opensea	chr10	133892406	133892567	3
0.0573	1.9250E-09	CMR59		IGR	opensea	chr7	129649151	129649196	2
-0.0571	7.3131E-09	CMR60	SLC6A16	TSS200	island	chr19	49828658	49828667	2
-0.0568	5.2999E-08	CMR61	SYCP2L	TSS1500	shore	chr6	10886573	10886967	2
0.0562	1.3401E-08	CMR62	IQCE	Body	shore	chr7	2646782	2647414	5
0.0557	4.6995E-08	CMR63	CCDC102B	5'UTR	opensea	chr18	66389420	66389447	2
0.0555	3.3118E-08	CMR64	CMC1	TSS1500	shore	chr3	28281945	28282245	2
0.0554	1.4900E-08	CMR65		IGR	shelf	chr6	169564460	169565259	2
-0.0554	4.7473E-08	CMR66	LOC84931	TSS200	opensea	chr2	121223947	121224009	3
0.0551	7.3695E-12	CMR67	GPR124	Body	opensea	chr8	37672607	37672725	2
-0.0550	3.5858E-08	CMR68	NKAIN3	TSS1500	island	chr8	63161077	63161676	6
-0.0548	2.8443E-10	CMR69	CPEB1	Body	island	chr15	83315922	83316223	2
0.0530	2.1109E-09	CMR70	RASA3	Body	opensea	chr13	114829170	114829496	2
0.0524	3.0390E-09	CMR71	TRIM31	Body	opensea	chr6	30079524	30079536	2
-0.0522	2.2167E-08	CMR72	NDUFS7	Body	island	chr19	1387894	1387991	2
-0.0520	3.3662E-10	CMR73	ANO1	TSS200	island	chr11	69924390	69924724	2
-0.0518	4.8156E-11	CMR74	SLC6A3	Body	opensea	chr5	1412236	1413138	2
-0.0516	1.7391E-08	CMR75	LRRC52	1stExon	opensea	chr1	165513699	165513716	2

0.0515	2.0062E-11	CMR76	RINT1	Body	opensea	chr7	105182555	105182968	2
0.0515	5.2722E-09	CMR77	EDARADD	TSS1500	shore	chr1	236557670	236558142	3
0.0515	2.7443E-09	CMR78	LPCAT1	Body	opensea	chr5	1518133	1518179	2
-0.0513	2.9004E-13	CMR79	SGPP2	TSS200	island	chr2	223289277	223289560	3
0.0512	2.3879E-12	CMR80		IGR	opensea	chr16	85006502	85007113	2
-0.0510	6.3981E-12	CMR81	YWHAE	TSS1500	island	chr17	1303810	1303864	2
0.0510	2.3476E-09	CMR82	EAPP	TSS1500	shore	chr14	35009492	35009523	3
0.0506	1.8497E-09	CMR83	TMEM246	TSS1500	shore	chr9	104249643	104249936	6
-0.0506	4.5724E-09	CMR84	FUT11	Body	shore	chr10	75533350	75533431	3
-0.0501	2.1273E-09	CMR85	PCDHGA4	Body	shore	chr5	140762229	140764485	11

Table S2. Results of mRNA expression of the selected genes

**Gene**	**fold change MEAN**		**p value**
	**MAST**	**CONT**	
ABCA2	1,05059702	1,09472707	0,491
SLC2A14	1,00959309	1,63626564	0,185
CDH7	-	-	-
TET2	2,58627005	2,3795486	0,403
DNTM3A	0,93547386	1,67946959	0,658
TPSB2	1,7867782	1,16602146	0,804
EAPP	1,17225194	1,07140954	0,752
EDARADD	0,99148009	1,09568898	0,528
GRM2	2,42735293	1,29805674	0,013
HDAC9	1,38911158	1,27632233	0,899
KRTCAP3	1,82219047	1,34761903	0,036
OTX2	-	-	-
RAB22A	1,51356827	1,02778243	0,483
RASGEF1	1,31683775	1,04720982	0,967
RUNX1	1,06108958	1,02786376	0,372

SCG2	-	-	-
SETD2	1,37415588	1,0786928	0,119
SGMS1	1,03709648	1,03287142	0,76
SH3PXD2	1,86620573	1,12833791	0,128

Table S3 a-c.

Tabela 3. The cell decomposition in the studied groups using three different methods: the Houseman method implemented in RefBaseEWAS,Table 3a; Robust Partial Correlations , Table 3b, and Cibersort (CBS), Table 3c.

Table S3a. The cell decomposition in the studied groups using three different methods: the Houseman method implemented in RefBaseEWAS

STUDY GROUP	CD8T	CD4T	NK	Bcell	Mono	Gran
CONT	0.07267264	0.1483493	0.06610037	0.03250597	0.07992596	0.5953870
MAST	0.05156082	0.1520580	0.03502721	0.03468947	0.06289315	0.6589662

Table S3b. The cell decomposition in the studied groups using Robust Partial Correlations

STUDY GROUP	CD8T	CD4T	NK	Bcell	Mono	Gran
CONT	0.07936191	0.1430781	0.07361965	0.04276324	0.07928041	0.5818967
MAST	0.06117826	0.1461951	0.04349404	0.04523343	0.06259314	0.6413061

Table S3c. The cell decomposition in the studied groups using Cibersort (CBS)

STUDY GROUP	CD8T	CD4T	NK	Bcell	Mono	Gran
CONT	0.07190795	0.1404726	0.06873357	0.04180945	0.08023738	0.5968391
MAST	0.05516744	0.1410714	0.03917300	0.04545167	0.06390287	0.6552336
